# Novelties in the Anatomy of the Central Nervous System and Related Disorders

**DOI:** 10.1155/2014/514974

**Published:** 2014-06-15

**Authors:** Branislav Filipović, Nevena Radonjic, Igor Jakovcevski, Milos Petrovic

**Affiliations:** ^1^Faculty of Medicine, Institute of Anatomy “Niko Miljanic”, University of Belgrade, Belgrade, Serbia; ^2^Faculty of Medicine, Institute of Medical and Clinical Biochemistry, University of Belgrade, Belgrade, Serbia; ^3^Neuroscience Department, University of Connecticut Health Center, Farmington, CT, USA; ^4^Center for Molecular Neurobiology, Universitätsklinikum Hamburg-Eppendorf, Hamburg, Germany; ^5^Cellular Neurobiology and Neurophysiology Laboratories, Centro de Investigación Médica Aplicada y Universidad de Navarra, Pamplona, Spain

Anatomy of the central nervous system (CNS) is far beyond the classical approach to anatomy investigations. Studies of the CNS are, nowadays, comprising various fields of medicine, starting from clinical investigations, up to the animal models, so they are useful in studies of different neuropsychiatric diseases and disorders, such as schizophrenia, posttraumatic stress syndrome, and Alzheimer's disease. Serious investigations in the field of the CNS are inseparable from the technological advances in the research, but also in diagnostics. This is the reason why we have gathered, at a glance, the papers that have very little in common. But the* file rouge* is the anatomy of the CNS and its changes illustrated in this unity in differences. Our initial aim was to illustrate the importance of the knowledge of CNS anatomy, for either the investigators in this field or the clinicians, devoted to solving the problem of serious neuropsychiatric diseases.

Two papers are dealing with the effects of maternal deprivation on rats brains. Maternal deprivation represents widely used animal model of schizophrenia and it is based on the 24 hr separation of the 9-day-old offspring from their mother. The rats were sacrificed in early adult period. Dr. M. Aksić and his team revealed that maternally deprived rats had a 28% smaller cell soma area in the prefrontal cortex, (30% in retrosplenial cortex), and 15% in motor cortex compared to the controls. Using the same model, Dr. B. Marković and coauthors outlined the significant decrease in the activity of acetylcholine esterase in the cortex and increase in the hippocampus of maternally deprived rats.

Dr. M. Kutová and coworkers are claiming that gross* postmortem* changes in the planum temporale in Alzheimer's disease may be at least partially attributed to neurohistological changes in the layer III pyramidal neurons.

An interesting study of hypothalamopituitary-adrenocortical role in the hyperthermia has been conducted by Tseng et al. Their results suggest that human umbilical cord blood-derived stem cells therapy may improve outcomes of heatstroke in mice by reducing systemic inflammation as well as hypothalamopituitary-adrenocortical axis impairment.

A clinical study of posttraumatic stress disorder (PTSD), presented by Dr. A. Starcevic et al., demonstrates a significant reduction in the volumes of amygdala in the left hemisphere in the PTSD patients, suggesting the eventual role of amygdaloid complex in this disease.

All presented papers show the interest of the investigators of different kinds in the anatomy of the CNS and its change in different conditions, in the animals in the experimental models,* postmortem* human brains, or PTSD patients. With the advances in knowledge of the CNS anatomy structures, perhaps their role in the pathogenesis of diseases and disorders of different origin is going to be, even partially, clarified.

*Branislav Filipović* *Nevena Radonjic* *Igor Jakovcevski* *Milos Petrovic*

